# Physicians’ Perceptions of Somatic Complaints Among Bedouin Arab Women in Polygamous and Monogamous Marriages

**DOI:** 10.3390/healthcare14050633

**Published:** 2026-03-02

**Authors:** Alean Al-Krenawi, Avital Laufer

**Affiliations:** 1 Resilience Research Centre, Dalhousie University, Halifax, NS B3H 4R2, Canada; alean@bgu.ac.il; 2 Spitzer Department of Social Work, Ben-Gurion University of the Negev, Beer-Sheva 8410501, Israel; 3 School of Behavioral Sciences, Netanya Academic College, Netanya 4210002, Israel

**Keywords:** somatization, polygamous, monogamous, family, physicians, well-being

## Abstract

**Background:** Women in polygamous marriages are known to experience higher levels of psychological distress. However, less is known about how physicians perceive and interpret their complaints and how these perceptions shape the patient–physician relationship. **Aim:** This study aimed to examine physicians’ perceptions of somatic complaints among Bedouin Arab women in polygamous versus monogamous marriages and to explore differences in the patient–physician relationship. **Methods:** This quantitative study included 126 participants—75 women in monogamous marriages and 51 in polygamous marriages—along with their treating physicians. Women completed self-report measures assessing psychological distress, self-esteem, marital satisfaction, and family functioning. Physicians provided information regarding clinic utilization patterns, symptom characteristics, and their interpretations of patients’ complaints. **Results:** Women in polygamous marriages reported significantly higher psychological distress, greater somatization, poorer family functioning, and reduced marital satisfaction compared to women in monogamous marriages, consistent with previous research. Physicians reported more frequent unscheduled clinic visits among women in polygamous marriages and were more likely to interpret their complaints as having psychological rather than purely physical origins. These patterns reflected differences in clinical perceptions and patient–physician interactions between the groups. **Conclusions:** The findings highlight differences in how physicians perceive and respond to complaints presented by women in polygamous versus monogamous marriages. These results underscore the importance of culturally informed clinical approaches and increased awareness of potential biases in primary healthcare settings.

## 1. Introduction

In societies where polygamous marriages are prevalent, women encounter distinct social and emotional challenges that can significantly impact their well-being. Extensive research has shown that women in polygamous marriages often experience higher levels of psychological distress and lower marital satisfaction compared to their counterparts in monogamous relationships. However, there is limited understanding of how these women’s concerns are perceived and interpreted by healthcare providers. This understanding of the potential disconnect between patient and physician perceptions is particularly important in the Bedouin Arab community of southern Israel, where cultural norms, gender roles, and traditional family structures shape both women’s experiences and their access to healthcare.

Somatization is broadly conceptualized as the tendency to express psychological distress through physical symptoms that lack sufficient medical explanation [1,2]. In primary care settings, somatization constitutes a significant diagnostic and treatment challenge, frequently resulting in repeated visits, extensive medical workups, and patient–provider frustration [3]. Psychological and cultural frameworks emphasize that somatization is influenced not only by individual vulnerability and emotional distress but also by family dynamics, social norms, and culturally organized practices of emotional expression [4,5].

In many non-Western societies, including Arab communities, somatic complaints may serve as a socially acceptable way to communicate distress, particularly when verbalizing psychological suffering is stigmatized [6]. Linguistic and conceptual frameworks, such as the Arabic concept of *tajseed* (“embodiment”), underscore a holistic, non-dualistic view of mind and body, in contrast to the Western biomedical distinction between psychological and physical symptoms [7].

In marginalized minority groups, physician bias may exacerbate these challenges. The patient–physician encounter is shaped by implicit cultural assumptions on both sides. Patients may expect the physician to legitimize their suffering through physical explanations, while physicians, especially those trained in Western biomedical frameworks, may emphasize psychological interpretations when symptoms lack clear physical foundations. These mismatches can lead to miscommunication, dissatisfaction, and repeated clinic visits [8].

In societies undergoing rapid modernization and social transformation, cultural idioms of distress may shift, becoming more complex and multifaceted. This dynamic is particularly relevant for Bedouin society in southern Israel, where transitions in lifestyle, education, gender roles, and economic structures coexist with strong traditional norms.

### Bedouin Arab Society in Israel

The Bedouin population in the Negev comprises a unique Indigenous minority characterized by tribal affiliation, collectivist social organization, and firm adherence to traditional cultural values [9,10]. Historically semi-nomadic, the community has undergone rapid sedentarization and modernization over recent decades, often amid profound socioeconomic disadvantages.

Polygamy continues to be prevalent in this community, despite being prohibited under Israeli civil law, with an estimated 20% of marriages involving polygamous arrangements, even among the younger generation [11]. Marriages within the Bedouin population are often conducted in accordance with Islamic religious law (*Sharia*) rather than through the civil registry, resulting in a disconnect between legal documentation and actual practices. The Israeli authorities have historically implemented a policy of limited enforcement in minority communities, particularly concerning family matters deemed part of religious and cultural autonomy [12]. As a result, there is minimal enforcement of the prohibition on polygamous marriages within the Bedouin community.

In the Bedouin society, women’s lives are shaped by patriarchal norms that restrict mobility, decision-making, and public participation. Women are expected to prioritize family obligations, adhere to modesty expectations, and maintain family honor through socially regulated behavior [13]. Despite these constraints, Bedouin women often hold significant informal influence within the domestic sphere, managing households, negotiating kin relationships, arranging marriages, and serving as custodians of cultural knowledge through storytelling and ritual practices [14].

These gendered dynamics have important implications for healthcare utilization. Because social and physical mobility are limited, primary healthcare clinics frequently serve as accessible and culturally acceptable spaces where women can seek care, social interaction, and emotional support. For some women, clinic visits may fulfill both medical and social needs, contributing to patterns of high visitation rates [15].

Extensive research conducted across Middle Eastern, African, and Southeast Asian societies consistently shows that women in polygamous marriages experience poorer mental health outcomes compared to those in monogamous marriages. These women report higher levels of psychological distress, somatic symptoms, lower self-esteem, and diminished family functioning [16,17,18,19,20]. Factors such as chronic stress, marital inequity, and reduced emotional support among polygamous families contribute not only to psychological symptoms but also to the physical expression of distress [21,22]. While these patterns are well documented, little is known about how physicians perceive and respond to such complaints in the Bedouin context and how these perceptions interact with women’s subjective experiences.

Therefore, the primary aim of this study was to examine gaps between physicians’ perceptions and women’s reports of their complaints in polygamous versus monogamous marriages. A secondary aim was to compare psychological distress between the two groups to replicate previous findings.

The hypotheses of the study were: A. Women in polygamous marriages will report higher levels of psychological distress, lower self-esteem, poorer family functioning, and reduced marital satisfaction compared to women in monogamous marriages. B. Physicians will more frequently attribute complaints of women from polygamous marriages to psychological causes than those of women from monogamous marriages.

## 2. Method

### 2.1. Participants

**Women.** The target population included Bedouin Arab women living in monogamous or polygamous marriages who visited the participating primary healthcare clinics. To ensure the study captured women with frequent clinic utilization, eligibility required at least two clinic visits per week.

The initial sampling plan targeted 200 women (100 monogamous, 100 polygamous). Due to time constraints and challenges in reaching eligible participants, the final sample included 75 women from monogamous families (60%) and 51 women from polygamous families (40%). The total sample was 126 women. Participants were selected consecutively during clinic visits, with efforts made to include women across a range of ages and socioeconomic backgrounds.

**General practitioners** (GPs) working at the clinics were recruited to complete a brief questionnaire for each participating woman. All participating GPs were licensed family physicians who were familiar with the patient population. A total of seven general practitioners participated in the study: four male and three female. In terms of ethnicity, four practitioners identified as Arab, while three identified as Jewish. None of the practitioners approached declined to participate.

### 2.2. Ethical Considerations

The study received approval from the institutional review boards (IRBs) of the authors’ affiliated universities. All participants provided informed consent before data collection. Participants were informed that their participation was voluntary, that they could withdraw at any time, and that their medical care would not be affected by refusal to participate. Confidentiality was emphasized, and participants were assured that no identifying information would be included in the study.

### 2.3. Procedure

Data were collected in two primary healthcare clinics located in Bedouin villages in southern Israel. These clinics serve as central and accessible points of medical care for residents. Data collection occurred over six months. Trained female data collectors who shared the cultural and linguistic background of the participants approached women in waiting rooms or immediately following consultations. Interviewers explained the purpose of the study, confidentiality procedures, and the voluntary nature of participation.

Depending on the participant’s preference, interviews were conducted either privately in a separate room or in a quiet area near the clinic. For women with limited reading or writing skills, interviewers read all questions aloud and recorded responses verbatim.

Following each woman’s interview, data collectors approached her GP to complete a matched physician-report questionnaire. Physicians typically completed these forms during or immediately after the clinical encounter.

### 2.4. Measures

**Sociodemographic Variables**. The questionnaire included questions on age, type of marriage (monogamous or polygamous), number of children, women’s occupation, years of education, and perceived socioeconomic status (1 = very poor to 6 = excellent).

**Self-esteem**. Self-esteem was measured by the Rosenberg [23]. Self-Esteem Scale, which consists of 10 questions ranging from 1 to 4, with higher scores indicating higher self-esteem. The reliability of the translated Arabic questionnaire among Bedouin women was examined in a previous study [5] and was found to be good (α = 0.79). In this study, similar reliability was found for the measure (α = 0.71).

**Family Functioning** was assessed using the General Functioning subscale of the McMaster Family Assessment Device [24,25], which includes 12 items rated from 1 (healthy) to 4 (poor functioning). The reliability in this study was α = 0.73. This shortened version has been validated in prior studies with Bedouin families [5].

**Marital Satisfaction** was measured with a 10-item abbreviated version of the Dyadic Adjustment Scale [26]. A 5-point Likert scale is used, with higher scores indicating lower satisfaction. The reliability in this study was high (α = 0.94). This shortened scale has demonstrated strong psychometric properties in Bedouin samples [5].

**Psychological Symptomatology** was assessed using the SCL-90-R [27,28]. The scale has nine symptom dimensions: somatization, obsessive-compulsive, interpersonal sensitivity, depression, anxiety, hostility, phobic anxiety, paranoid ideation, psychoticism, and a Global Severity Index (GSI). Reliability coefficients in this study ranged from α = 0.77 to 0.92, and overall GSI α = 0.98. The Arabic version of the SCL-90-R was used among various Arab populations, including Bedouin women, and was found to be valid [29].

**Physician-Report Questionnaire** was completed by each participant’s GP, assessing: frequency of clinic visits, whether visits were scheduled or unscheduled, primary reason for visit (physical, mental, or both), physician’s perception of core problem, patient’s level of agreement with diagnosis (1—low to 9—high), reported pain level (1–9), and level of complaints (1–9). This instrument was specifically developed for the current study to capture clinically relevant aspects of healthcare utilization and symptom presentation. The questionnaire was constructed based on consultations with experienced family physicians who routinely work with Bedouin patients.

### 2.5. Data Analysis

Data was analyzed with SPSS version 29. Descriptive statistics were used with demographic and background variables. *t*-tests and Chi-square tests were used to examine differences between women from monogamous vs. polygamous families regarding their self-esteem, family functioning, and marital satisfaction. In addition to *p*-values, effect sizes and confidence intervals (95% CI) were computed for all group comparisons. Degrees of freedom for each test are reported in the results tables. Statistical significance was set at *p* < 0.05, with emphasis on effect sizes and clinical relevance.

## 3. Results

Differences between the groups regarding sociodemographic variables are presented in Table 1. The women in monogamous marriages were younger, more educated, and had fewer children than did the women in the polygamous families. Monogamous families were shown to experience better economic conditions than polygamous families. The economic status was generally rated as moderate. This means that the respondents believed their economic situation was the same as that of the rest of the population, neither higher nor lower.

Differences between the women from monogamous vs. polygamous families regarding their self-esteem, family functioning, and marital satisfaction are presented in Table 2. The findings indicate that women in monogamous marriages reported better family functioning and more marital satisfaction than their polygamous counterparts.

Table 3 presents differences in the somatization variables. The results indicate that the women from polygamous families suffered significantly more than the women from monogamous families in all the examined dimensions.

Table 4 presents physicians’ reports on women’s health clinic visits, including frequency, main complaints, and agreement with the physician’s diagnosis. The results indicate that the women in polygamous marriages visited the healthcare clinics more frequently than the women in monogamous marriages. Approximately three-quarters of the polygamous group arrived at the clinic without a prior appointment, whereas less than half of the monogamous group did so. A higher proportion of women from polygamous marriages sought medical attention due to pain. The frequency of visits to the GP for fever or fatigue was similar in both groups.

According to the GPs’ observations, most women in monogamous marriages visited the clinic solely for physical reasons. In contrast, about one-fifth of the visits by women in polygamous marriages were due to mental health reasons, and an additional 23% of the visits were due to a combination of both. According to the GPs, the polygamous group tended to express more complaints and reported more severe and intense pain than the monogamous group. In addition, the GPs reported that the monogamous group exhibited a significantly higher level of agreement with the GPs’ diagnoses than did the polygamous group.

## 4. Discussion

The primary aim of the present study was to examine perceptual discrepancies between general practitioners (GPs) regarding complaints presented by Bedouin women in polygamous versus monogamous marriages. The findings reveal substantial differences in patterns of clinic utilization, symptom interpretation, and psychological functioning between the two groups. Women in polygamous marriages demonstrated significantly higher clinic utilization, including more frequent unscheduled visits, compared to their monogamously married counterparts. Their physicians were more likely to attribute these women’s complaints to psychological rather than physical causes. Moreover, women in polygamous marriages expressed lower agreement with their physicians’ diagnoses. Consistent with these perceptions, they also reported higher levels of psychological distress and greater family-related difficulties. These disparities appeared in both self-reported measures and physician assessments, highlighting a significant perceptual gap during clinical encounters. Consequently, our two hypotheses were validated.

As expected by our first hypothesis and consistent with prior literature, women in polygamous marriages reported higher levels of somatization, depression, anxiety, and overall psychological distress, alongside poorer family functioning and reduced marital satisfaction. These findings replicate earlier studies indicating that polygamous marital structures are associated with chronic emotional strain [16,17,18,19,20,21,22]. Importantly, our study extends this body of knowledge by demonstrating that these disparities are not confined to self-reported distress but are also reflected in physicians’ clinical perceptions and diagnostic reasoning.

Family dynamics within polygamous households may be particularly salient in shaping somatization processes, given the complex relational structures, power asymmetries, and chronic interpersonal stressors that often characterize these families. Research on polygamous family systems has documented heightened levels of marital conflict, competition between co-wives, emotional neglect, and limited spousal availability, all of which may constrain direct emotional expression and coping [21]. Within such contexts, somatic symptoms may function as an indirect and socially legitimate mode of expressing distress. Somatization can thus be understood not solely as an individual response but as embedded in relational patterns through which emotional strain is managed and communicated in polygamous family structures.

As for our second hypothesis, as expected, when assessing their GPs, we found asymmetry in how GPs interpret women’s complaints across marital contexts. Physicians were significantly more likely to attribute symptoms reported by women in polygamous marriages to psychological or psychosomatic causes, whereas complaints presented by women in monogamous marriages were more readily framed as physical. This pattern aligns with theoretical models of clinician attribution bias, which suggest that providers may unconsciously rely on social categorizations when interpreting symptoms [30]. Such biases are particularly salient in cross-cultural healthcare settings, where minority patients’ somatic complaints are more likely to be psychologized [31].

It is important to consider an alternative interpretation of these findings. Women in polygamous marriages reported significantly higher levels of psychological distress across all symptom domains. This suggests that physicians’ tendency to attribute their complaints to psychological causes may reflect accurate clinical judgment rather than bias in attribution. However, even if physicians are accurate in their assessments, our findings indicate that this recognition does not necessarily translate into perceived support or effective care from the patients’ perspective. Women in polygamous marriages reported significantly lower agreement with their physicians’ diagnoses, which indicates dissatisfaction with the clinical encounter. This discrepancy suggests that although GPs may correctly identify emotional suffering, they may lack the culturally appropriate tools necessary to address these issues in a way that feels meaningful and responsive to these women. This is especially true in societies where psychological difficulties are not discussed but embodied through physical complaints. From this viewpoint, lower agreement with physicians’ diagnoses may reflect a mismatch between biomedical–psychological explanatory models and patients’ culturally grounded understandings of distress. Therefore, accurately identifying distress is insufficient unless it is accompanied by culturally sensitive interventions, appropriate referrals, and communication strategies that resonate with the patients’ lived experiences.

Communication gaps may increase the risk of misinterpretation, potentially obscuring underlying medical needs and reinforcing disparities in care [30,32]. Physician-reported outcomes are embedded within the clinical relationship and may be influenced by prior expectations. The finding that women in polygamous marriages reported less agreement with their GPs’ diagnoses may contribute to a breakdown in effective communication and hinder the establishment of a strong patient–provider relationship, leading to worse treatment for these women.

The findings are further illuminated by cross-cultural models of somatization. Western biomedical paradigms typically maintain a sharp distinction between physical and psychological domains. In contrast, non-Western cultural frameworks conceptualize distress holistically [33]. Kleinman [34] argued that somatic idioms of distress represent culturally meaningful ways of communicating suffering, particularly in contexts where direct emotional expression is constrained. Within Bedouin society, where women, especially in polygamous arrangements, may experience restricted autonomy, interpersonal conflict, and social marginalization, somatic complaints may function as a socially legitimate channel for expressing distress [16,17].

The study results suggest that women seek psychological and social support at nearby primary clinics, as was found in other studies [15]. However, physicians in these clinics, who already have heavy workloads, may overlook non-physiological factors when patients present with complaints that seem to have no clear physical cause. This may prevent the physicians from offering alternative solutions that could provide relief to these women. This dynamic may help explain not only the nature of the clinical encounters but also the broader patterns of healthcare utilization observed in this population. When psychosocial needs remain insufficiently addressed during routine visits, women may continue to return to primary care settings in search of support, thereby shaping distinctive patterns of service use.

From a healthcare utilization standpoint, GPs reported significantly higher clinic attendance and unscheduled visits among women in polygamous families. Prior research suggests that frequent primary care visits often function as informal help-seeking for emotional distress [3,4]. In under-resourced communities, clinics may represent accessible spaces for social support. However, unscheduled utilization is widely recognized as a marker of inefficiency and system strain, contributing to provider burnout and reduced continuity of care.

Importantly, these patterns suggest that unmet psychosocial needs translate into disproportionate and potentially preventable pressure on healthcare systems. This highlights a critical implication for healthcare policy and practice: improving culturally responsive care may reduce unnecessary utilization while enhancing patient support and medical outcomes.

### 4.1. Implications for Clinical Care

Given the heavy loads carried by primary care physicians in Bedouin communities, complaints lacking clear medical explanations may not always receive adequate exploration of underlying psychosocial factors. Improving physicians’ cultural competence, including awareness of how Bedouin women conceptualize distress, the role of traditional coping strategies, and cultural norms regarding communication, is essential to fostering more effective clinical interactions.

Culturally sensitive training for healthcare professionals should explicitly address family structures and gendered power dynamics that shape help-seeking, coping, and exposure to distress. Training programs should include focused modules on identifying context-specific risk factors, such as intra-family hierarchy, limited autonomy, and cumulative stressors. Concrete recommendations include: (a) case-based training using scenarios involving women from polygamous households to enhance clinical sensitivity to hidden distress; (b) skills development for conducting culturally attuned assessments that consider marital structure, social positioning, and access to support; and (c) collaboration with culturally knowledgeable mediators to prevent stereotyping while ensuring ethical and gender-responsive care. Integrating these components into both pre-service education and ongoing professional supervision may enhance clinicians’ ability to provide nuanced, equitable care in culturally complex and trauma-affected contexts.

By implementing such training, primary care settings can become more supportive environments through which women from polygamous families receive both medical and emotional care in a culturally grounded manner. This integrated approach has the potential to strengthen resilience, reduce repeated clinic visits driven by unaddressed psychological needs, and improve overall well-being.

### 4.2. Limitations

This study has several limitations. First, women in the monogamous and polygamous groups differed significantly in age, education, employment, economic status, and number of children. These sociodemographic disparities may have contributed to differences in psychological distress and somatization and should be regarded as potential confounding variables that limit causal interpretation and the generalizability of the results. Additionally, the study did not assess other potentially relevant factors such as wife order (first, second, and third wife) or duration of polygamous marriage, which may influence women’s mental health and help-seeking behaviors.

The criterion for participation was at least two clinic visits per week. This may have resulted in selection bias of women with higher symptom severity or stronger health-seeking behaviors. As a result, the sample may not fully represent women with milder symptoms or those who face barriers to frequent clinic attendance. Consequently, the generalizability of the results to broader community samples or to women with lower treatment utilization may be limited. Additionally, the sample size, while adequate for exploratory analyses, may limit statistical power. It is also important to remember that this study is cross-sectional; therefore, no causal inferences can be made. Future studies should include longitudinal or qualitative follow-up to deepen understanding of these complex psychosocial processes.

The study notably emphasizes the perceptions of general practitioners (GPs), which may be shaped by personal biases or cultural assumptions regarding polygamy. These perceptions could influence diagnostic reasoning and treatment choices, ultimately impacting the findings of the research itself. Moreover, the use of a study-specific, non-standardized physician questionnaire hinders the comparability of our findings with those of other studies and may impact the reliability and validity of the reported clinical assessments. Additionally, due to institutional data retention policies and the unavailability of the raw dataset, we were unable to perform further statistical analyses or implement more advanced multivariate models. This limitation constrains our ability to account for potential confounding variables and to thoroughly investigate the robustness of the observed group differences.

## 5. Conclusions

The dual-source methodology, integrating women’s self-reports with physicians’ clinical assessments, offers a more comprehensive perspective on the lived experiences of women in polygamous marriages and on the complex interrelations among psychological, somatic, and relational dimensions. Nevertheless, the findings must be interpreted with careful consideration of the study’s methodological constraints. Although women in polygamous marriages exhibited higher levels of psychological distress and distinctive patterns of healthcare utilization, these differences cannot be attributed exclusively to marital structure. In light of the pronounced sociodemographic disparities between the groups, including age, educational attainment, employment status, and economic conditions, the results should be understood as correlational associations rather than evidence of causal effects related to marriage type.

Accordingly, the observed patterns most likely reflect a multifaceted interaction between marital context and broader structural, cultural, and social determinants. This underscores the importance of strengthening culturally sensitive practices within primary healthcare, particularly when serving marginalized minority populations. Training programs for family physicians working with Bedouin communities should therefore emphasize culturally informed communication, reflexive clinical awareness, and sensitivity to culturally shaped illness narratives. Such an approach may enhance diagnostic accuracy, foster more effective physician–patient relationships, and reduce recurrent consultations linked to somatization, ultimately contributing to more equitable and responsive healthcare delivery.

## Figures and Tables

**Table 1:** Demographic characteristics of women in monogamous and polygamous marriages.

m (sd)	Total (*n* = 126)	Monogamous (*n* = 75)	Polygamous (*n* = 51)	*t* (df = 124)
Age	32.33 (1.77)	27.71 (6.43)	41.48 (9.76)	−9.30 ***
range	(18–65)	(18–47)	(26–65)	
Number of children	4.16 (3.17)	3.21 (2.13)	5.51 (3.85)	−4.23 ***
Socioeconomic status (1 = worst, 6 = excellent)	3.55 (1.29)	3.86 (1.32)	3.08 (1.09)	3.42 ***
				χ^2^
Women working	*n* = 58 (46.4%)	*n* = 39 (53%)	*n* = 19 (37%)	(df = 1) 3.86 *
Not working	*n* = 67 (53.6%)	*n* = 35(47%)	*n* = 32 (63%)	
Years of education:				(df = 2) 23.76 ***
Up to 10 y	*n* = 41 (33%)	*n* = 12 (16%)	*n* = 29 (56%)	
10–12 y	*n* = 48 (38%)	*n* = 36 (48%)	*n* = 12 (24%)	
12 y and up	*n* = 37 (29%)	*n* = 27 (36%)	*n* = 10 (20%)	

* *p* < 0.05, *** *p* < 0.001.

**Table 2:** Differences in Self-Esteem, Family Functioning, and Marital Satisfaction between Women in Monogamous and Polygamous Marriages.

m (sd)	Total (*n* = 126)	Monogamous (*n* = 75)	Polygamous (*n* = 51)	*t* (df = 124)
Self-esteem (1 = low to 4 = high)	3.08 (0.50)	3.11 (0.59)	3.05 (0.41)	0.70
Family functioning (1 = low level of problems to 4 = high level of problems)	2.84 (0.48)	2.69 (0.42)	2.95 (0.49)	−3.05 **
Marital satisfaction (1 = high satisfaction to 5 = low satisfaction)	2.33 (1.17)	1.87 (0.91)	3.01 (1.18)	−6.17 ***

** *p* < 0.01, *** *p* < 0.001.

**Table 3:** Group Differences in Somatization and Psychological Symptom Dimensions among Women in Monogamous and Polygamous Marriages (SCL-90-R Subscales).

m (sd)	Total (*n* = 126)	Monogamous (*n* = 75)	Polygamous (*n* = 51)	*t* (df = 124)
Somatization	1.26 0.98)	0.91 (0.81)	1.77 (0.98)	−5.36 ***
Obsessive-compulsive	1.29 (0.90)	0.98 (0.84)	1.74 (0.80)	−5.05 ***
Interpersonal sensitivity	1.17 (0.96)	0.89 (0.92)	1.57 (0.86)	−4.13 ***
Depression	1.19 (0.91)	0.92 (0.88)	1.60 (0.79)	−4.18 ***
Anxiety	1.15 (0.89)	0.85 (0.78)	1.59 (0.87)	−4.99 ***
Phobic anxiety	0.86 (0.78)	0.63 (0.69)	1.19 (0.79)	−4.23 ***
Hostility	1.02 (0.90)	0.77 (0.79)	1.39 (0.94)	−4.03 ***
Paranoid ideation	1.13 (0.90)	0.84 (0.84)	1.56 (0.81)	−4.77 ***
Psychoticism	1.19 (0.44)	1.07 (0.36)	2.76 (0.78)	−3.75 ***
GSI	0.86 (0.79)	0.62 (0.71)	1.21 (0.77)	−4.42 ***

*** *p* < 0.001.

**Table 4:** General Practitioners’ Observations of Healthcare Utilization and Symptom Presentation among Women in Monogamous and Polygamous Marriages.

	*n*, %	Total (*n* = 126)	Monogamous (*n* = 75)	Polygamous (*n* = 51)	χ^2^
	Weekly visit	30, 24%	8, 11%	22, 43%	
Frequency of visits	Monthly visit	52, 42%	32, 43%	20, 39%	(df = 2) 21.02 ***
	Less than a monthly visit	43, 34%	34, 46%	9, 18%	
Type of visit	Scheduled	51, 43%	38, 53%	13, 27%	(df = 1) 7.78 **
	Not scheduled	69, 57%	34, 47%	35, 73%	
	High fever	9%	8%	10%	
Reason for the visit	Fatigue	11%	11%	12%	(df = 3)
	Pain	41%	36%	49%	23.52 ***
	Other	39%	45%	29%	
	Physical	58%	84%	22%	(df = 3)
The core of the problem	Mental	8%	1%	18%	51.75 ***
	Both physical and mental	23%	11%	23%	
	Other	11%	3%	22%	
	m (sd)				*t* (df = 124)
Agreement with GP’s diagnosis 1 = disagreement to 9 = high agreement		8.23 (1.46)	8.67 (0.76)	7.60 (1.92)	4.24 ***
Level of pain 1 = low to 9 = high		4.54 (0.86)	3.96 (0.45)	5.33 (1.2)	4.61 ***
Level of complaints 1 = low to 9 = high		4.50 (0.72)	3.79 (0.63)	5.49 (1.31)	5.24 ***

** *p* < 0.01, *** *p* < 0.001.

## Data Availability

The data presented in this study are available from the corresponding author upon reasonable request. This limitation arises from authors’ collaboration agreement with Maccabi Healthcare Services, which requires us to notify them of any third-party data sharing.

## References

[1] Lipowski Z.J. (1988). Somatization: The Concept and Its Clinical Application. Am. J. Psychiatry.

[2] De Gucht V., Fischler B. (2002). Somatization: A Critical Review of Conceptual and Methodological Issues. Psychosomatics.

[3] Kroenke K. (2003). Patients Presenting with Somatic Complaints: Epidemiology, Psychiatric Comorbidity and Management. Int. J. Methods Psychiatr. Res..

[4] Clarke D.M., Piterman L., Byrne C.J., Austin D.W. (2008). Somatic Symptoms, Hypochondriasis and Psychological Distress: A Study of Somatization in Australian General Practice. Med. J. Aust..

[5] Al-Krenawi A., Graham J.R. (2004). Somatization among Bedouin Arab Women: Differentiated by Marital Status. J. Divorce Remarriage.

[6] Kleinman A.M. (1977). Depression, Somatization, and the New Cross-Cultural Psychiatry. Soc. Sci. Med..

[7] Al Busaidi Z.Q. (2010). The Concept of Somatisation: Cross-Cultural Perspective. Sultan Qaboos Univ. Med. J..

[8] Lehmann M., Pohontsch N.J., Zimmermann T., Scherer M., Löwe B. (2021). Diagnostic and Treatment Barriers to Persistent Somatic Symptoms in Primary Care. BMC Fam. Pract..

[9] Al-Krenawi A., Graham J.R., Al-Krenawi S. (1997). Social Work Practice with Polygamous Families. Child Adolesc. Soc. Work J..

[10] Alhuzail N.A. (2018). The social representation of the Bedouin woman. Women’s Stud. Int. Forum.

[11] Alhuzail N.A., Besser A., Zeigler-Hill V. (2024). Sharing Your Husband: Adult Attachment Styles and Emotional Responses of Israeli Bedouin Arab Women to Potential Polygynous Marriage. Int. J. Environ. Res. Public Health.

[12] Ramadan M.A. (2005). The Shari’a in Israel: Islamization, Israelization and the invented Islamic law. UCLA J. Islam. Near EL.

[13] Al-Krenawi A. (2000). Bedouin Arab Clients’ Use of Proverbs in the Therapeutic Setting. Int. J. Adv. Couns..

[14] Quader S.A., Oplatka I. (2008). The Power of Femininity: Exploring the Gender and Ethnic Experiences of Muslim Women Who Accessed Supervisory Roles in a Bedouin Society. J. Educ. Adm..

[15] Loignon C., Dupéré S., Benhadj L., Carru D., Dahrouge S. (2022). Perspectives of Structurally Marginalised Patients Attending Contextually Tailored and Integrated Care Practices in Canada. BMJ Open.

[16] Al-Krenawi A. (2012). A Study of Psychological Symptoms, Family Function, Marital and Life Satisfactions of Polygamous and Monogamous Women: The Palestinian Case. Int. J. Soc. Psychiatry.

[17] Al-Krenawi A. (2013). Mental Health and Polygamy: The Syrian Case. World J. Psychiatry.

[18] Shepard L.D. (2013). The Impact of Polygamy on Women’s Mental Health: A Systematic Review. Epidemiol. Psychiatr. Sci..

[19] Bahari I.S., Norhayati M.N., Hazlina N.H.N., Aiman C.A.A.M.S., Arif N.A.N.M. (2021). Psychological Impact of Polygamous Marriage on Women and Children: A Systematic Review and Meta-Analysis. BMC Pregnancy Childbirth.

[20] Rahmanina P., Khadeeja M., Firdaus M., Choudhry F.R. (2021). Prevalence of Mental Health Problems in Women in Polygamous versus Monogamous Marriages: A Systematic Review and Meta-Analysis. Arch. Womens Ment. Health.

[21] Al-Krenawi A., Graham J.R. (2006). A Comparison of Family Functioning, Life and Marital Satisfaction, and Mental Health of Women in Polygamous and Monogamous Marriages. Int. J. Soc. Psychiatry.

[22] Al-Krenawi A., Lightman E. (2000). Learning Achievements, Social Adjustment and Family Conflicts among Bedouin Arab Children from Polygamous and Monogamous Families. J. Soc. Psychol..

[23] Rosenberg M. (1979). Components of Rosenberg’s Self-Esteem Scale. Conceiving the Self.

[24] Epstein N.B., Baldwin M.N., Bishop D.S. (1983). The McMaster Family Assessment Device. J. Marital. Fam. Ther..

[25] Miller I.W., Epstein N.B., Bishop D.S., Keitner G.I. (1985). The McMaster Family Assessment Device: Reliability and Validity. J. Marital. Fam. Ther..

[26] Lavee Y., McCubbin H., Olson D. (1987). The Effect of a Stressful Life Event and Transitions in Family Functioning and Well-Being. J. Marriage Fam..

[27] Derogatis L.R. (1992). SCL-90-R Manual: Administration, Scoring, and Procedures.

[28] Derogatis L.R. (1977). SCL-90-R Administration, Scoring, and Procedure Manual for the Revised Version.

[29] Al-Krenawi A., Graham J.R., Sehwail A.M. (2002). Bereavement Responses among Palestinian Muslim Widows, Sons, and Daughters Following the Hebron Massacre. Omega.

[30] van Ryn M., Burke J. (2000). The Effect of Patient Race and Socio-economic Status on Physicians’ Perceptions of Patients. Soc. Sci. Med..

[31] Kirmayer L.J., Young A. (1998). Culture and Somatization: Clinical, Epidemiological, and Ethnographic Perspectives. Psychosom. Med..

[32] Street R.L., Makoul G., Arora N.K., Epstein R.M. (2009). How does Communication Heal? Pathways Linking Clinician-Patient Communication to Health Outcomes. Patient. Educ. Couns..

[33] Kirmayer L.J., Narasiah L., Munoz M., Rashid M., Ryder A.G., Guzder J., Hassan G., Rousseau C., Pottie K. (2011). Common Mental Health Problems in Immigrants and Refugees: General Approach in Primary Care. Can. Med Assoc. J..

[34] Kleinman A. (1988). The Illness Narratives: Suffering, Healing, and the Human Condition.

